# Upregulation of α7 Nicotinic Receptors by Acetylcholinesterase C-Terminal Peptides

**DOI:** 10.1371/journal.pone.0004846

**Published:** 2009-03-16

**Authors:** Cherie E. Bond, Martina Zimmermann, Susan A. Greenfield

**Affiliations:** 1 Institute for the Future of the Mind, Department of Pharmacology, Oxford University, Oxford, United Kingdom; 2 Department of Pharmacology, Johann Wolfgang von Goethe University, Frankfurt am Main, Germany; Vrije Universiteit Amsterdam, Netherlands

## Abstract

**Background:**

The alpha-7 nicotinic acetylcholine receptor (α7-nAChR) is well known as a potent calcium ionophore that, in the brain, has been implicated in excitotoxicity and hence in the underlying mechanisms of neurodegenerative disorders such as Alzheimer's disease. Previous research implied that the activity of this receptor may be modified by exposure to a peptide fragment derived from the C-terminal region of the enzyme acetylcholinesterase. This investigation was undertaken to determine if the functional changes observed could be attributed to peptide binding interaction with the α7-nAChR, or peptide modulation of receptor expression.

**Methodology/Principal Findings:**

This study provides evidence that two peptides derived from the C-terminus of acetylcholinesterase, not only selectively displace specific bungarotoxin binding at the α7-nAChR, but also alter receptor binding properties for its familiar ligands, including the alternative endogenous agonist choline. Of more long-term significance, these peptides also induce upregulation of α7-nAChR mRNA and protein expression, as well as enhancing receptor trafficking to the plasma membrane.

**Conclusions/Significance:**

The results reported here demonstrate a hitherto unknown relationship between the α7-nAChR and the non-enzymatic functions of acetylcholinesterase, mediated independently by its C-terminal domain. Such an interaction may prove valuable as a pharmacological tool, prompting new approaches for understanding, and combating, the process of neurodegeneration.

## Introduction

Excitotoxicity, due to excessive and deleterious calcium influx into cells, has long been recognised as a common mechanism underlying the range of neurodegenerative diseases including Alzheimer's, Parkinson's and Motor Neuron diseases [1]–[4]. One of the most powerful calcium ionophores in the brain is the alpha-7 nicotinic acetylcholine receptor (α7-nAChR; CHRNA7) [5]. Due to its highly selective calcium ion (Ca^2+^) permeability, the α7-nAChR not only facilitates transmitter release via activation of inhibitory or excitatory Ca^2+^-sensitive ion channels, but also triggers calcium signalling cascades that can initiate gene transcription, influence axonal pathfinding, and mediate apoptotic cell death [6]–[9].

The α7-nAChR receptor has already been implicated directly in Alzheimer's disease, in that it binds amyloid-beta (Aβ) peptide [10]. In addition, α7-nAChR expression levels are altered in relevant mouse models of Alzheimer's disease [11]–[13], as well as in various tissues from human patients [14]–[16]. Furthermore, the most effective therapeutic agents for Alzheimer's disease, such as the anti-acetylcholinesterase drug galantamine, also target the α7-nAChR [17]–[18]. However unlike its other cholinergic counterparts, the α7-nAChR can be activated by a primary ligand other than acetylcholine, ie. choline [19]–[20]. Hence the α7-nAChR can function in areas of the brain devoid of cholinergic transmission *per se*, where the far more ubiquitous choline may act as a substitute ligand. This phenomenon is particularly relevant in the light of reports that Alzheimer's disease could be linked to an aberration in choline uptake mechanisms, independent of the cholinergic synapse [21]–[23].

**Figure 1:**
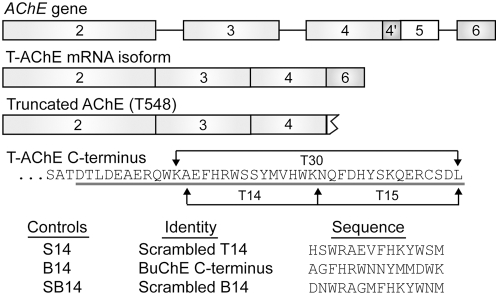
AChE and control polypeptides used in this study. All isoforms of AChE are derived from a single gene transcript and contain the invariable exons 2, 3 and 4. The T-AChE isoform arises through alternative mRNA splicing of exon 6 to the invariable exons. Truncated AChE (T548) is a recombinant protein, translated from cDNA containing exons 2, 3, and 4, but lacking a C-terminal exon. The underlined amino acid sequence highlights the unique C-terminus of the T-AChE isoform derived from exon 6 of the *AChE* gene with the location and sequence of AChE peptides indicated. Control peptides used in experimentation include: S14, a scrambled version of AChE T14 peptide; B14, comprising the 14 amino acid region in butyrylcholinesterase (BuChE) that is homologous to AChE T14; and SB14, the scrambled version of the same region of BuChE.

Similarly, it has been proposed that acetylcholinesterase (AChE, EC 3.1.1.7) could have non-enzymatic functions unrelated to the cholinergic synapse [2], [24]–[26]. Previous evidence, albeit indirect, has indicated that a 14-amino acid peptide sequence in the C-terminus of AChE independently modulates α7-nAChR responses to agonists [27]–[28]. This peptide bears a striking homology and structural similarity to Aβ [2] and replicates [29]–[33] many of the non-hydrolytic actions now well established for the enzyme in a wide range of preparations and particular situations, such as development [25]–[26], [34]–[36] and apoptosis [37]–[38]. However, recent detailed analyses of critical amino acid residues [39]–[40], potential protease cleavage sites in the C-terminus of AChE [41], and structural features [39]–[41], have now identified a larger candidate bioactive motif of 30 amino acids that encompasses the originally recognized 14 amino acid sequence (Fig. 1). Since both peptides are derived from the “tailed” (T) isoform of AChE and are comprised of 14 and 30 amino acids respectively, for convenience they are referred to as ‘T14’ and ‘T30’.

The aim of this study was to compare, *in vitro*, the direct effects of T14 and T30 in displacement of radioligand binding of the α7-nAChR antagonist, α-bungarotoxin (α-BTX), with a number of scrambled or related peptides serving as controls (Fig. 1). Moreover, we considered the effects of peptide modulation of the binding properties of known α7-nAChR ligands, including and especially, choline. In addition to investigating the effects of the peptides on receptor binding parameters, we also explored possible peptide involvement in regulation of the receptor. Accordingly, we measured the actions of chronic T14 and T30 peptide exposure on α7-nAChR mRNA and protein expression, as well as subcellular localization of the receptor.

## Results

### Peptide-induced changes in α7-nAChR binding

**Figure 2:**
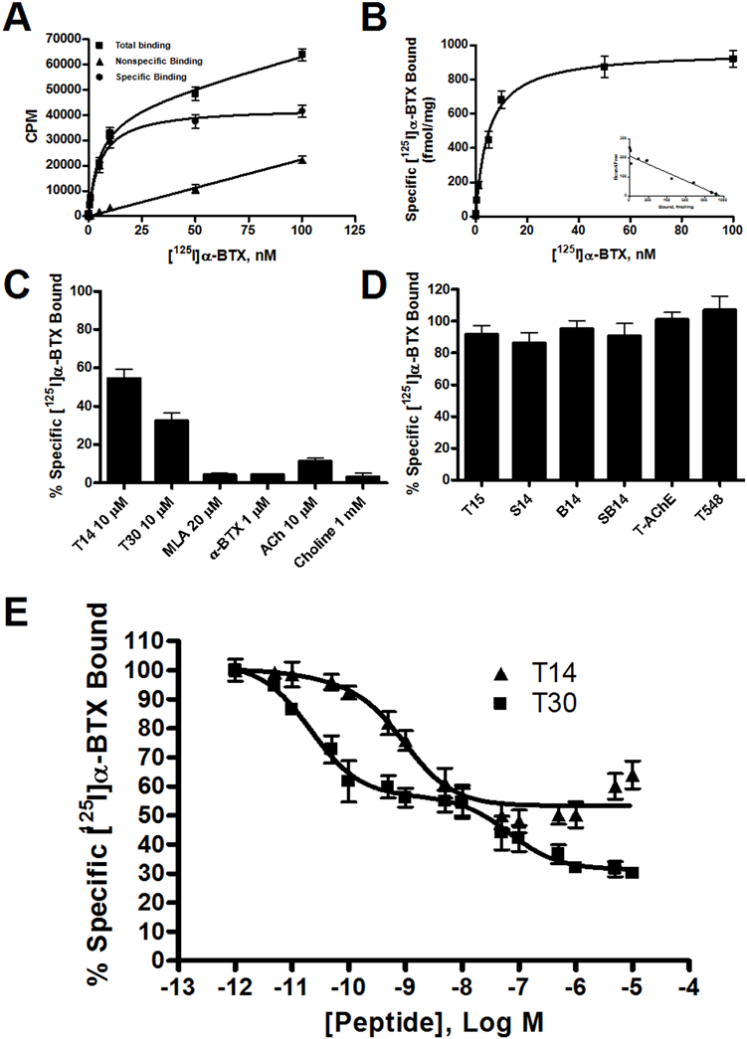
Acute live cell binding in GH4-hα7 cells. Non-specific binding was determined in the presence of 10 µM methyllycaconitine (MLA). Data shown are the average±SEM of 3 separate experiments. A. Raw saturation binding data shows total, nonspecific, and specific binding for [^125^I]α-BTX to α7-nAChR in this cell line. B. Specific binding of [^125^I]α-BTX to α7-nAChR in fmol/mg protein with Scatchard analysis. C. Comparison of maximal specific [^125^I]α-BTX binding displacement by AChE peptides T14 and T30, as compared with known α7-nAChR antagonists and agonists. ACh = acetylcholine. D. Maximal specific [^125^I]α-BTX binding displacement by control peptides, full length T-AChE, and truncated AChE (T548). E. Displacement binding profiles for AChE C-terminal peptides T14 and T30.

Initial experiments were performed to characterize the binding parameters of the human α7-nAChR heterologously expressed in the rat GH4-hα7 cell line using a live-cell binding method. Saturation binding was carried out with [^125^I]alpha-bungarotoxin ([^125^I]α-BTX) concentrations ranging from 0.03 nM to 100 nM. Non-specific binding was determined in the presence of 10 µM methyllycaconitine (MLA). Total radioligand bound was consistently <10% of free radioligand in the assay. High levels of concentration-dependent and saturable specific [^125^I]α-BTX binding in this cell line were observed (Fig. 2A, 2B). The high correlation coefficient of hyperbolic curve fitting (R^2^ = 0.9775) is consistent with binding to a single class of receptors. Binding parameters were established as B_max_ = 965.8±28.9 fmol/mg protein; K_d_ = 4.68±0.61 nM (Mean±SEM). Saturation binding repeated at intervals throughout the experimental period verified that the levels observed initially were maintained through multiple passages (up to 30), demonstrating the stable nature of α7-nAChR expression by this cell line.

Preliminary screening of the binding of both peptides to the α7-nAChR, when compared with that of acknowledged receptor agonists and antagonists, revealed significant, but incomplete, competition with [^125^I]α-BTX for receptor binding sites (Fig. 2C). T14 exhibited approximately 40% efficacy at 10 µM concentration, whereas the same concentration of T30 was 70% efficacious. Higher concentrations of these peptides did not further displace radioligand binding. In contrast, none of the control peptides were able to compete with [^125^I]α-BTX for binding to the α7-nAChR (Fig. 2D). Similarly, neither full-length T-AChE, nor truncated T548, had an effect on [^125^I]α-BTX binding to the receptor (Fig. 2D).

To elucidate further T14 and T30 binding parameters, full displacement binding profiles were performed (Fig. 2E). Interestingly, a two-phased activity was observed. First, a high affinity binding was apparent that fit the classic one-site competition model expected for displacement from a single class of receptors. T14 and T30 were equally efficacious at the high affinity site, with maximum displacement at about 45% of total specific binding, however T30 (K_i_ = 16.8±1.8 pM) displayed much greater potency than T14 (K_i_ = 653.3±12.6 pM). A second, lower affinity site was identified for T30 that accounts for a further 25% binding displacement by the peptide (K_i_ = 47.1±2.8 nM). Comparative analysis of the data for T30, using Akaike's Information Criteria method, confirmed that the two-site competition model fits the data better than a one-site competition model with a >99.99% probability that it is correct. In contrast, for T14, although a one-site competition model provided an acceptable fit to the data in the 1 pM to 10 nM range, with increasing concentrations of peptide >10 nM, a reversal of displacement efficacy was observed.

**Figure 3:**
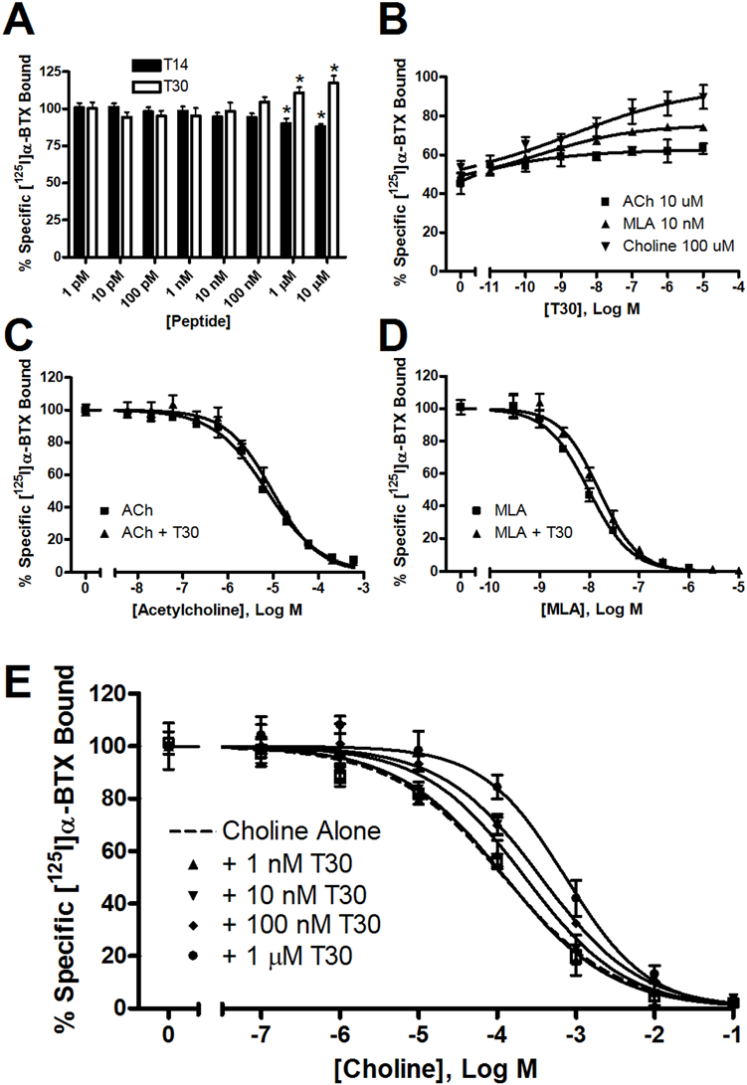
Acute membrane binding in GH4-hα7 cells. Data shown are the combined results of a minimum of 2 experiments each performed in triplicate and expressed as percent control specific binding±SEM. A. Competition binding with T14 and T30 concentrations varying from 1 pM to 10 µM. B. Effect of varying concentrations of T30 on α-BTX competition binding with ACh, MLA, and choline. C. Competition binding curve for ACh vs. ACh+T30 (100 nM). D. Competition binding curve for MLA vs. MLA+T30 (100 nM). E. Competition binding curve for choline vs. choline+T30 at various concentrations of T30.

We then appraised peptide binding to α7-nAChR in purified membrane preparations. Unexpectedly, neither T14, nor T30, had a significant effect on [^125^I]α-BTX binding to GH4-hα7 cell membranes in the 1 pM to 100 nM range (Fig. 3A). As compared with control maximum specific binding values, a statistically significant increase in [^125^I]α-BTX binding was observed in the presence of 1 µM (Mean±SEM = 110.9±3.6%, p = 0.0271) or 10 µM (117.3±4.9%, p = 0.0172) T30. Conversely, T14 displayed a small but significant displacement of [^125^I]α-BTX binding at high concentrations (1 µM = 90.11±3.5%, p = 0.0378; 10 µM = 87.91±1.9%, p = 0.0014).

To explore the possibility that T30 might act through an allosteric site to affect binding of other α7-nAChR ligands to the receptor, as has been seen for T14 previously in functional studies, varying concentrations of T30 were incubated with cell membranes in the presence of methyllycaconitine (MLA), acetylcholine (ACh), or choline at constant concentrations equivalent to their measured EC50 values (Fig. 3B). Specific binding displacement was altered by T30 in a concentration-dependent manner, with significant decreases in specific binding efficacy of 18% for ACh (p = 0.0426), 24% for MLA (p = 0.0032), and 36% for choline (p = 0.0064) as compared with the individual ligands alone.

Subsequently displacement binding was performed for each ligand in the presence and absence of a constant concentration of T30 (100 nM). Global fitting analysis was performed to compare whole binding curve differences. A small but statistically significant (p = 0.032) rightward shift was observed for ACh+T30 (IC50 = 9.5±0.5 µM) as compared with ACh alone (IC50 = 7.2±0.3 µM; Fig. 3C). In the presence of T30, the binding profiles for MLA and choline were similarly shifted to the right, though to a greater degree than that seen for ACh. Comparison of binding parameters revealed a highly significant (p = 0.006) decrease in competitive potency for MLA+T30 (IC50 = 15.5±0.7 nM) as compared with MLA alone (IC50 = 9.5±0.4 nM; Fig. 3D). Since the effect of T30 on choline binding was greater than that seen for either ACh or MLA, we expanded the experimental method to examine the effect of a range of T30 concentrations on choline binding profiles. As shown in Fig. 3E, we observed a highly significant (p<0.0001) concentration-dependent decrease in choline competitive potency in the presence of T30. Comparative IC50 and K_i_ values are shown in Table 1. K_i_ was calculated from the IC50 using the equation of Cheng and Prusoff [42] based on a constant radioligand concentration of 2 nM with a K_d_ = 4.68 nM.

**Table 1:** Comparison of EC50 and Ki values showing the effect of increasing concentrations of T30 on choline binding to the α7-nAChR.

Ligand	IC50±SEM (µM)	K_i_±SEM (µM)
Choline alone	122.9±7.4	85.9±5.1
+T30 1 nM	126.0±12.8	88.9±8.9
+T30 10 nM	225.0±21.2	157.3±16.9
+T30 100 nM	357.4±33.0	249.9±24.5
+T30 1 µM	736.6±68.2	515.1±44.6

**Figure 4:**
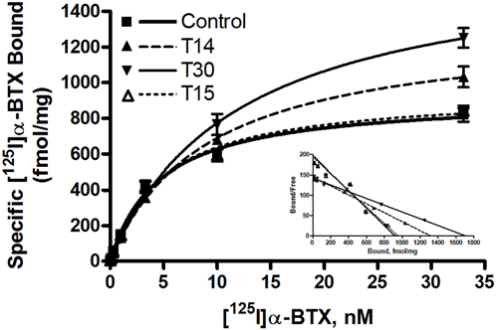
Specific saturation binding on cell membranes after chronic treatment of GH4-hα7 cells with 100 nM T-AChE peptides for 24 hr. Data shown are the average±SEM of 2 separate experiments each performed in triplicate.

We next examined the effects of chronic peptide exposure on the number of α7-nAChR receptor binding sites and receptor affinity for [^125^I]α-BTX binding (Fig. 4). Cells in culture were exposed to AChE peptides for 24 hr, then saturation binding assays were performed on purified cell membranes using [^125^I]α-BTX in concentrations ranging from 0.033 to 33.0 nM. After 24 hr treatment with T14 or T30, a significant increase (p = 0.0004 and p<0.0001 respectively) in the number of α7-nAChR binding sites, as determined by maximal binding values, was observed (Fig. 4). Additionally, the affinity of receptors for [^125^I]α-BTX was significantly decreased (T14, p = 0.0035; T30, p = 0.0018) as compared with controls. In contrast to that seen for T14 and T30 peptides, T15 treatment for 24 hr had no effect on specific binding affinity of [^125^I]α-BTX to the α7-nAChR or on the number of available receptor binding sites (Fig. 4). Average B_max_ and K_d_ values for α7-nAChR binding after chronic peptide exposure are summarized in Table 2.

**Table 2:** Summary of saturation binding parameters showing the effects of chronic T-AChE peptide treatment on the number of α7-nAChR binding sites (B_max_) and receptor affinity (K_d_) for α-BTX.

24 hr Treatment	B_max_ (fmol/mg)±SEM	K_d_ (nM)±SEM
Control	918±33.5	4.66±0.54
T14	1313±50.9	9.06±0.93
T30	1704±81.1	12.01±1.39
T15	949±33.0	4.91±0.53

### Peptide-induced changes in α7-nAChR mRNA expression

**Figure 5:**
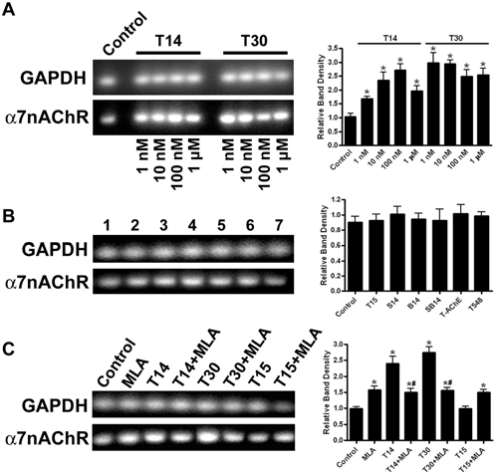
RT-PCR analysis of mRNA expression in GH4-hα7 cells after 24 hr exposure to peptides. GAPDH expression was used as an internal standard. Gels shown are representative results of experiments; accompanying graphs provide semi-quantitative data determined from a minimum of 3 separate experiments. Asterisks (*) indicate values statistically different from controls. A. Effect of varying concentrations of AChE peptides T14 and T30 on α7-nAChR expression. B. Effects of 100 nM control peptides, 10 nM full-length T-AChE, or 10 nM truncated T-AChE on α7-nAChR expression. Lane 1 = Control, 2 = T15, 3 = S14, 4 = B14, 5 = SB14, 6 = full-length T-AChE, 7 = truncated T-AChE (T548). C. Effects of MLA (10 µM) on peptide-induced changes in α7-nAChR expression in cells treated with 100 nM T14, T30, or T15. Hash marks (#) indicate peptide+MLA values significantly different from peptide alone values.

To assess the effects of T-AChE C-terminal peptides on α7-nAChR mRNA expression, total cellular RNA was isolated and analysed for gene-specific expression levels using reverse transcriptase PCR. Specifics of primer design and sequences used in RT-PCR experiments are described in the methods. RT minus controls were negative and gene expression in control cells did not change noticeably throughout the series of experiments. Expression of the housekeeping gene glyceraldehyde 3-phosphate dehydrogenase (GAPDH) was used as a standard for comparative analysis. RT-PCR analysis was performed in control GH4-hα7 cells and those exposed to AChE peptides at concentrations ranging from 1 nM to 1 µM for 24 hr (Fig. 5A). After T14 or T30 peptide treatment, α7-nAChR mRNA expression was significantly upregulated for all concentrations of the peptides tested as compared with controls: T14 1 nM 1.74±0.08 (relative band density±SEM), p = 0.0225; 10 nM 2.35±0.29, p = 0.0272; 100 nM 2.72±0.22, p = 0.0168; 1 µM 1.96±0.19, p = 0.0411; T30 1 nM 2.98±0.37, p = 0.0341; 10 nM 2.94±0.14, p = 0.0055; 100 nM 2.49±0.24, p = 0.0263; 1 µM 2.54±0.24, p = 0.0241. Levels of α7-nAChR mRNA displayed a concentration-dependent increase with T14 treatment, with maximal expression at 100 nM. A similar high level of α7-nAChR expression was achieved after treatment with only 1 nM T30. As peptide concentrations increased further, α7-nAChR expression levels remained appreciably enhanced as compared with controls at all peptide concentrations tested.

To test whether the increased expression observed was directly attributable to sequence specific T-AChE peptide interaction with the receptor, rather than random non-specific peptide effects or structural motif interaction, cells were similarly treated with control peptides, followed by analysis of α7-nAChR mRNA expression. No significant change in α7-nAChR mRNA levels was observed after exposure to T15 (p = 0.5893), S14 (p = 0.3124), B14 (p = 0.4331) or SB14 (p = 0.7378) peptides (Fig. 5B). In addition, neither the full length T-AChE molecule (p = 0.3419), nor the truncated T548 (p = 0.1778), effected a significant change in α7-nAChR mRNA expression.

We next attempted to block peptide-induced α7-nAChR mRNA enhancement using the α7-nAChR specific inhibitor MLA. Exposure of GH4-hα7 cells to 10 µM MLA for 24 hr also induced significant upregulation of α7-nAChR expression (1.58±0.12, p = 0.0399) as compared with controls, although to a lesser degree than did 100 nM T14 (2.61±0.23, p = 0.0269) or T30 (2.74±0.19, p = 0.0117) peptides (Fig. 5C). When MLA and T14 (1.50±0.12) or T30 (1.56±0.09) were co-applied however, the greater enhancement of peptide-induced α7-nAChR expression was suppressed to levels observed after MLA treatment alone. Treatment for 24 hr with T15 had no effect on α7-nAChR mRNA expression (p = 0.9446) and T15 co-applied with MLA was non-different from MLA treatment alone (1.50±0.09). Values for T14 or T30 peptide treatments alone were significantly different than peptide+MLA as determined by Tukey's multiple comparison test (p<0.01 and p<0.001 respectively), whereas T15 vs T15+MLA was not (p>0.05).

### Peptide-induced changes in α7-nAChR protein expression

**Figure 6:**
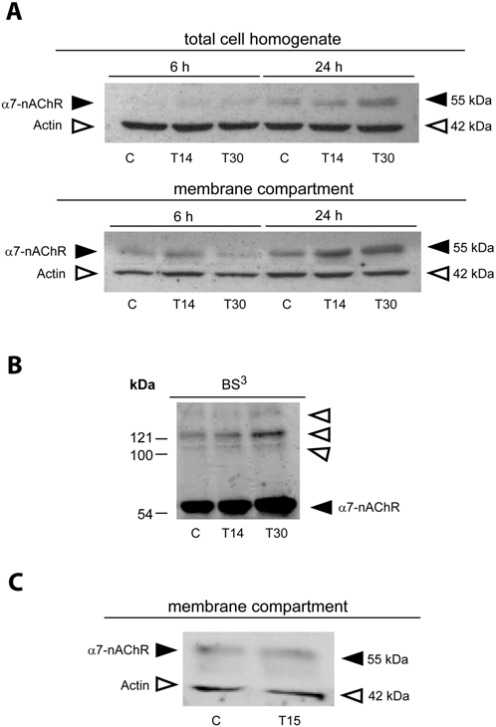
Representative western blots of α7-nAChR protein levels in control (C) and peptide (T14, T30) treated GH4-hα7 cells. All experiments were performed a minimum of 2 times. A. Protein levels as assessed in total cell homogenate and in membrane compartments after 6 or 24 hr peptide exposure. The filled and open arrow heads indicate α7-nAChR and actin at 55 and 42 kDa MW, respectively. Actin was used as an internal standard. B. After 24 hour peptide treatment, cells were treated with the membrane-impermeant cross-linking reagent bis(sulfosuccinimidyl)suberate sodium salt (BS^3^). The filled and open arrow heads indicate α7-nAChR and high molecular weight aggregated species of the α7-nAChR receptor, respectively. C. Representative western blot of α7-nAChR protein levels in control and T15 (control peptide) treated cells.

To examine AChE peptide effects on protein expression, α7-nAChR protein levels were analyzed by SDS-PAGE and Western blot. A slight, but discernable, upregulation of α7-nAChR protein expression was detected after only 6 hr exposure to 100 nM T14 or T30 in both total cell homogenates and purified membrane fractions (Fig. 6A, filled arrowheads). After 24 hr treatment with peptides, a profound increase in α7-nAChR protein levels was observed. This increase was particularly pronounced in the membrane fractions of cells treated with the AChE peptides for 24 hr. Furthermore, T30 treatment induced a greater increase in receptor protein levels at 24 hr than did T14.

To determine whether the induced increase in α7-nAChR protein levels in the membrane compartment reflects an upregulation of receptor protein at the plasma membrane, peptide-treated GH4-hα7 cells were exposed to the cross-linking agent BS^3^ prior to harvesting for analysis. As can be seen in Fig. 6B, T14 and T30 both increase α7-nAChR levels in the membrane compartment. In addition, higher amounts of high molecular weight aggregated species of the α7-nAChR were observed for T30 incubated cells as compared with controls (Fig. 6B, empty arrowheads. In order to verify that BS^3^ does not permeate cell membranes, and thus exclude the possibility that observations were confounded by cross-linked intracellular α7-nAChR protein, actin levels were also assessed in BS^3^-treated cells. The immunoreactivity for this intracellular protein was unaffected by the BS^3^ treatment (data not shown). In contrast to that seen for T14 and T30, treatment of the cells for 24 hours with the control peptide T15 had no effect on α7-nAChR levels in cell membranes (Fig. 6C).

**Figure 7:**
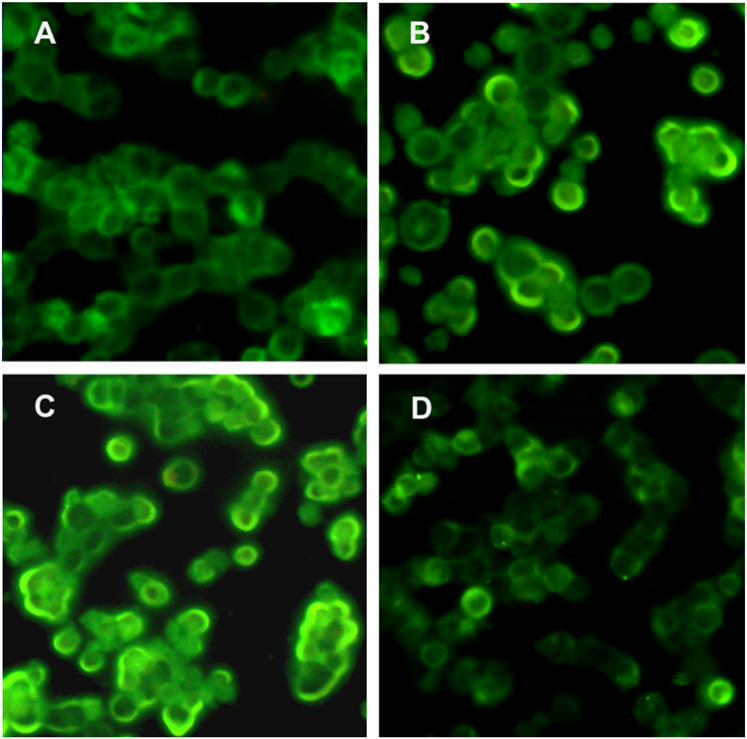
Representative photographs of immunofluorescent staining for α7-nAChR in GH4-hα7 cells. A. Control cells. B. Cells pretreated for 24 hr with 100 nM T14. C. Cells pretreated for 24 hr with 100 nM T30. D. Cells pretreated for 24 hr with 100 nM T15.

In addition, we examined changes in α7-nAChR protein further using immunofluorescent staining. High levels of α7-nAChR protein were detected in cellular membranes, but not in cytoplasmic or perinuclear regions (Fig. 7). Background control cells incubated with secondary antibodies, but lacking primary antibodies, did not produce a discernable signal (data not shown). After treatment for 24 hr with T14 (Fig. 7B) or T30 (Fig. 7C), enhanced signal intensity was evident in cell membranes as compared with controls (Fig. 7A), with T30-treated cells showing the greatest increase. In contrast, T15-treated cells were similar in appearance to control cells (Fig. 7D).

## Discussion

### Technical Considerations

In general, heterologous expression of the α7-nAChR in non-neuronal mammalian cells has proven extremely problematic, at best producing cell lines with only transient or sporadic functional expression [43]. However the GH4-hα7 cell line (Merck & Co, Inc, Rahway, USA) chosen for this study, is a stable transfectant expressing high levels of the human α7-nAChR (Fig. 2B), as well as a full complement of native cell surface receptors and ion channels [44]. GH4 cells, derived from a rat pituitary tumor cell line, are widely established as a model for investigating mechanisms of intracellular calcium homeostasis and trophic factor release in secretory cells [45]. These cells also produce high levels of the chaperone protein RIC-3 [46], recently discovered to be essential for efficient folding, assembly, and functional expression of the α7-nAChR in mammalian cells [47]. Thus this cell line is capable of sustaining high levels of functional α7-nAChR expression and provides a good model system for study of ligand interactions with the α7-nAChR.

### Rationale for investigating the effects of peptides derived from T-AChE

For over a decade, it has been known that transient co-expression α7-nAChR and AChE are spatially and temporally correlated during development [48]–[50], where these molecules play an integral role in the regulation of neuronal proliferation and differentiation [34], [51], neurite outgrowth [26], and programmed cell death [52]–[53]. However, many of the prominent features of developmental processes are recapitulated in degenerative disease [2] and are similarly accompanied by changes in AChE and α7-nAChR expression [54]–[55]. More than two decades ago, Fossier and colleagues [56] first envisaged a hypothetical, yet direct, non-hydrolytic action of AChE causing upregulation of the AChR. Yet, although it is recognized that activation of α7-nAChR can up-regulate AChE expression [55], possible reciprocal regulation of the receptor by AChE, has never been investigated. This suggestion can now be explored more vigorously given current knowledge of the multiple molecular isoforms of AChE.

Over-expression of the major “tailed” isoform of AChE found in the adult brain (T-AChE) has been associated with neurodeterioration [57]–[58], and induction of apoptosis [59]. Furthermore, T-AChE accumulates in amyloid plaques, where it enhances Aβ fibril formation and exacerbates the neurotoxic effects of Aβ [60]–[62], in contrast to an alternative isoform, ‘readthrough’ AChE (R-AChE), which attenuates these characteristics, and displays neuroprotective effects following stress-induced upregulation [57], [61]. Yet these AChE isoforms have identical catalytic activity [63], differing only in their alternatively spliced C-termini [64]. Therefore the differences seen must be attributable to these unique domains of the proteins.

In parallel to the definitive findings established for R-AChE [61], [65], evidence is only now accumulating that the C-terminal domain of T-AChE may also undergo proteolytic cleavage *in vivo* [41], [66]–[69]. This helical domain independently exhibits autonomous bioactivity comparable to many of the non-catalytic effects attributed to the intact T-isoform of AChE [29]–[31], [33], [35], [39], [61], [66], [70]. Whilst the peptide fragment T14 retains some of the highly conserved residues essential to the functionally important elements of this domain, the longer T30 peptide contains more of the critical structural features required for disulfide bond formation and association with proline-rich domains, necessary for the formation of the many oligomeric states of AChE [39].Thus while T14 may comprise a minimal domain for association with the α7-nAChR, T30 exhibits more robust bioactivity and has potentially greater physiological relevance than T14.

### Binding of AChE peptides to the α7-nAChR

In live cell preparations, both T14 and T30 displaced [^125^I]α-BTX with high affinity in the picomolar to nanomolar range, comparable to that seen for Aβ [10]. However the similarity in the effects of the two peptides diverged at concentrations greater than 10 nM. While T30 appeared to act at a second site to displace further [^125^I]α-BTX binding, increasing concentrations of T14 caused an opposite effect. Since T30 is a much larger molecule than T14, it may contact additional allosteric sites on the receptor, or sites that are moved into proximity due to conformational changes induced by occupation of high affinity sites. Conceivably, increasing saturation of α7-nAChR subunits by T30 could promote either homomeric or heteromeric disulfide bond formation via the cysteine residues at the C-termini of the peptide molecules. Moreover, preservation of critical aromatic residues in T30 confers structural stability to the molecule [39], whereas, under physiological conditions and at high concentrations, T14 undergoes a conformational change that promotes beta sheet, fibril, and aggregate formation [71].

The dual binding profile observed with T14 reflects results from our previous functional studies with the peptide showing that low concentrations of T14 acutely potentiate neurite outgrowth and agonist activation of the α7-nAChR [27], [31]. On the other hand, high concentrations, or chronic treatment, with the peptide had opposite effects, blocking receptor activation by agonists [27] and inducing apoptotic cell death [30]. This dual trophic-toxic action, initiated by peptide-induced calcium influx, was sensitive to blockade by α7-nAChR blockers selectively, appeared to target an allosteric site, and competed with α7-nAChR agents such as ivermectin.

In fact, concentration-dependent bimodal activity characterizes ligand interactions with the α7-nAChR, where high and low ligand concentrations appear to act through temporally distinct mechanisms at numerous allosteric sites. For example, the α7-nAChR agonists choline and nicotine exhibit dual effects on receptor mediated activity, both activating receptors directly and inhibiting transmission through receptor desensitization [72]. Similarly, many of the AChE inhibiting drugs used in the treatment of Alzheimer's disease, such as tacrine, physostigmine, and galantamine, display concentration-dependent, dual modulation of agonist activation of the α7-nAChR [73]–[74]. Indeed, Aβ also shows a comparable concentration-dependent bimodal action, whereby picomolar concentrations activate α7-nAChR and increase its expression [54], whereas nanomolar concentrations block receptor activity [75]–[76].

In contrast to the effects seen for T14 and T30, the control peptides S14, T15, B14 and SB14 had no effect on [^125^I]α-BTX binding to the α7-nAChR, thus demonstrating the specificity of T14 and T30 peptides at this receptor. Similarly, neither T548, nor full-length T-AChE displaced [^125^I]α-BTX binding. Since T-548 lacks the domain proposed to interact with α7-nAChR, this truncated AChE molecule predictably does not exhibit an ability to bind to the receptor. Although full-length T-AChE does contain the critical C-terminal sequence, the intact protein may be too bulky to allow access to receptor binding sites. Alternatively, the tertiary structure of the full-length AChE may constrain the C-terminal region into a conformation that occludes residues necessary for α7-nAChR binding.

In order to reduce the number of confounding physiological variables associated with live cell cultures, peptide binding was assessed in purified membrane preparations, but surprisingly, no comparable displacement of [^125^I]α-BTX binding was detected. The differences observed between live cell and membrane binding may be due to dynamic mechanisms in live cells, as opposed to the static conditions that prevail in purified cell membranes, suggesting that intermediary factors or temporally dependent cellular processes could be involved in enabling peptide interaction with the α7-nAChR. In the live cell binding method, the cells were incubated with peptides in cell growth medium for two hours at 37°C. During that time, many different physiological processes could affect binding to the a7-nAChR. Extant intracellular pools of receptors may be mobilized to the cell surface, receptors may be internalized and degraded, modulating molecules may interact with receptors, or with other membrane components, or receptors may undergo conformational or state changes. In contrast, binding to isolated membranes was performed at 4°C in a buffered salt solution, independent of cytoplasmic proteins or cellular processes.

In contrast to results obtained with the peptides alone, co-application of T30 with ACh, MLA, or choline altered binding of these ligands to the α7-nAChR. This finding is consistent with the results of earlier functional studies on T14 bioactivity [27]–[28] and indicates that T30 similarly exerts its effects acutely through an allosteric mechanism. However, given the structural properties of these peptides, we cannot rule out the possibility that they directly disrupt the lipid-protein interface by insertion into the plasma membrane, thereby interfering physically with receptor conformation or local membrane integrity.

For all ligands tested, the presence of T30 caused a right-ward shift in the binding curve. Early work characterizing the binding properties of the α7-nAChR showed that modification of receptor thiol groups and cleavage of disulfide bonds, important in the manifestation of affinity state changes, decreases the binding affinity of the receptor for agonists by 10-fold, shifting the dose-response curve to the right [77]. Likewise, T30 may interact with thiol groups on the α7-nAChR to alter agonist binding to the receptor. Alternatively, this amphiphilic peptide may bind an allosteric site at the lipid-protein interface of the receptor, as has been shown for cholesterol [78] and the neurosteroid promegestone [79]. However, it is likely that T30 modulation of α7-nAChR activity will prove highly complex and may involve both steric and allosteric mechanisms, as has been reported for most α7-nAChR agonists and non-competitive antagonists studied to date [80].

The observation that T30 can alter choline binding to the α7-nAChR, is of particular interest, since choline can act as the primary endogenous ligand for the α7-nAChR during development of the nervous system [74] and in areas of the mature brain, where, paradoxically, both AChE and the α7-nAChR are highly expressed [48]–[49], but there is little or no acetylcholine [24]. Under pathological conditions, such as stroke, head trauma and Alzheimer's disease, neuronal choline, AChE, and α7-nAChR levels increase significantly [54]–[55], [81]. While choline activation of the α7-nAChR may be important for maintaining receptor-mediated Ca^2+^ homeostasis throughout the brain [81], dysregulation of choline metabolism could lead to excitotoxic Ca^2+^ imbalances and has been implicated in the selective neuronal vulnerability characterizing Alzheimer's disease [22]–[23], [82]. The modulation observed here, of choline binding to the α7-nAChR by T30, suggests another potential functional justification for the presence of AChE in tissues devoid of its familiar substrate.

Saturation binding analysis revealed that chronic treatment with T14 or T30, but not T15, increased the number of available receptor binding sites and altered receptor affinity for ligands. These effects are consistent with that reported for chronic activation by α7-nAChR agonists, such as nicotine, choline, carbachol, and Aβ [83]–[86]. However, while agonist-induced upregulation of α7-nAChR is generally accompanied by increased affinity of receptors for agonists [83], [87], chronic peptide exposure decreased receptor affinity for agonists and antagonists. Thus, although AChE C-terminal peptides and α7-nAChR agonists similarly up-regulate functional α7-nAChR expression, these newly synthesized, or altered, receptors may exhibit highly different activity states.

### Peptide-induced α7-nAChR mRNA expression

Consistent with results from saturation binding experiments, T14 and T30 peptides induced a marked increase in α7-nAChR mRNA expression. This effect was mitigated by co-application of the α7-nAChR antagonist MLA, indicating that the peptide-induced upregulation is the result of direct interaction of the peptides with the receptor. Meanwhile, peptide controls had no effect on α7-nAChR mRNA levels, further diminishing the possibility that the observed increase was due to non-specific peptide effects. In addition neither the full-length T-AChE, nor truncated T548, had an effect on α7-nAChR expression levels, suggesting that regulation of α7-nAChR transcriptional responses is yet another of the increasing number of effects that cannot be attributed to the catalytic activity of AChE.

The results obtained with T14 and T30 are similar to that observed generally for activation of the α7-nAChR by agonists such as nicotine and choline. For example, nicotine stimulates rapid Ca^2+^-dependent gene transcription through cfos induction [6], CREB phosphorylation, and MAP-kinase activation [8]. Furthermore, microarray analysis has shown that chronic exposure to nicotine can cause alteration of gene expression in over 160 genes [9]. Early reports indicated that nicotine-induced increases in α7-nAChR expression are dependent on newly synthesized receptors [84], in contrast to more recent evidence that suggests receptor upregulation by choline and nicotine may occur at the post-translational level [85]–[86]. While the mechanism by which nicotine exerts its effects is still in contention, the data presented here clearly show that chronic exposure to nanomolar amounts of T14 or T30 increases α7-nAChR expression at the mRNA level.

### Peptide-induced increase in α7-nAChR protein levels at the plasma membrane

Since changes in RNA expression are not necessarily reflected in equivalent alterations in protein levels, we subsequently analysed AChE peptide effects on protein expression by Western blot analysis and immunocytochemistry. After chronic exposure, both T14 and T30 induced an increase in receptor protein levels. Fractionation of the whole cell homogenates revealed that the changes in protein levels seen not only reflected a general increase in α7-nAChR in the cell as a whole, but that they were particularly associated with enhanced receptor levels in cellular membranes.

Because the purified membrane compartment is, however, composed of both intracellular organelle and cellular plasma membranes, localization of enhanced α7-nAChR protein levels to the membrane fraction does not definitively prove that the receptors are reaching the cell surface. By applying the non-membrane permeable crosslinker BS^3^, it was possible to differentiate intracellular membranes from the plasma membrane, since only externally accessible proteins are crosslinked by BS^3^. Our results demonstrated that the increase in receptor protein levels induced by chronic peptide treatment was associated specifically with the plasma membrane, accompanied by a marked increase in receptor aggregates, thus reflecting an increased number of receptor subunits cross-linked together, or with associated membrane proteins [88].

These findings in Western blots were further substantiated by immunofluorescent staining with α7-nAChR antibodies. After treatment with T14 or T30, increased signal intensity was apparent on cell surfaces, and, consistent with all other results, T30-treatment elicited the greatest response. Hence selective peptides derived from the C-terminus of AChE are capable of causing an increased proliferation in the number of α7-nAChR on the external surface of cells that express this receptor.

The most likely explanation for the observations is that interaction of these peptides with the α7-nAChR stimulates receptor auto-upregulation via Ca^2+^ signalling cascades. However, these results do not rule out the possibility that the peptides could also interact more directly with signalling molecules or transcription factors to modulate α7-nAChR expression, possibly through interaction with proline-rich domains [40]. Certainly a number of transcription factors contain such motifs [89], most notably those involved in apoptosis [90]. Interestingly, it has been shown that T-AChE is translocated to the nucleus upon initiation of apoptosis [59], whilst a nuclear form of AChE has been identified in endothelial cells [69]. Given that the presence of AChE in the nucleus, particularly in non-neuronal cells, precludes its classical role in neurotransmission, it is reasonable to speculate that this molecule contributes in some capacity to the regulation of transcriptional events. In this regard, it is particularly interesting to note that transgenic mice over-expressing T-AChE present with significantly increased levels of α7-nAChR mRNA and protein [91]. The data reported here demonstrate that chronic exposure to intact T-AChE does not elicit upregulation of α7-nAChR mRNA, as do the C-terminal peptides independent of the enzyme. This finding provocatively suggests that cleavage of the C-terminus may be a prerequisite for T-AChE-induced upregulation of α7-nAChR.

### Conclusions

In any event, these results demonstrate that a 30mer peptide, and to a lesser extent one of its 14mer derivatives, define a domain within the C-terminus of AChE that has the capacity for selective interaction with the α7-nAChR, not only binding to the α7-nAChR and altering its affinity for endogenous agonists, but also upregulating expression of the receptor itself. Given that activation of α7-nAChR reciprocally up-regulates AChE expression, a potential positive feedback loop may well coordinate the two molecules. Although there is only indirect evidence as yet that the C-terminal of T-AChE, or a peptide fragment thereof, exists naturally as a free peptide in the brain, of immediate relevance is the potential to use exogenously applied AChE peptides as modulators of α7-nAChR expression and function. As such, these peptides could serve as tools providing novel insights into the dynamics of a receptor seminal to neurodegeneration.

## Materials and Methods

All reagents were purchased from Sigma-Aldrich Co. Ltd., Poole, UK, unless otherwise noted. Disposables and cell culture plasticware were from Fisher Scientific, Loughborough, UK. T14, S14, B14, and SB14 peptides were custom synthesized by AnaSpec (San Jose, CA., USA) at >90% purity. T15 and T30 peptides were custom synthesized by Genosphere Biotechnologies (Paris, France) at >95% purity. All peptides were synthesized by fmoc methodology, purified by HPLC and analysed by mass spectrometry. Truncated T-AChE (T-548) was a gift from Palmer Taylor (Dept of Pharmacology, University of California, San Diego). [^125^I]α-bungarotoxin was purchased from GE Healthcare Bio-Sciences, Amersham, UK.

### Cell Culture

GH4-hα7 cells (Merck & Co., Rahway, USA) were maintained in Dulbecco's modified Eagle's medium (DMEM) with 4500 mg/l glucose and GlutaMAX (Life Technologies Ltd., Paisley, UK) containing 10% fetal bovine serum, 100 units/ml penicillin, 100 µg/ml streptomycin, 2.5 µg/ml amphotericin B, and the selective antibiotic, geneticin (G418; 500 µg/ml).

### Radioligand Binding Assays

For live cell binding experiments, cells were seeded into 6-well plates at a density of 1×10^5^ cells/well and allowed to recover for 24–48 hours before experimentation. Cells were treated with indicated peptides or α7-nAChR ligands for 30 min at 37°C in cell medium containing 1% FBS. Then [^125^I]α-bungarotoxin ([^125^I]α-BTX; 150 Ci/mmol) was added and cells were incubated at 37°C for a further 1.5 hr. Cell layers were washed 3× with 2 ml serum-free DMEM, then 0.5 ml 1 M NaOH was added to each well to lyse cells. Cell lysates were transferred to 5 ml scintillation fluid and radioactivity was determined using a Beckman LS6000IC scintillation counter.

For membrane binding experiments, confluent cells were scraped off 75 cm^2^ culture plates into ice-cold lysis buffer (20 mM Tris-HCl, pH 7.0, 5 mM ethylenediaminetetraacetic acid (EDTA), and 1× protease inhibitor cocktail (Roche Diagnostics, Ltd.,West Sussex, UK). After pelleting by centrifugation for 10 min at 13,000 rpm, cells were resuspended in 7 ml ice-cold lysis buffer, lysed by Dounce homogenization, and then centrifuged at 1000×g for 10 min. Supernatant was removed and the extraction process repeated. The supernatants were combined and centrifuged at 50,000 rpm (70 Ti rotor) for 30 min (Beckman Ultracentrifuge). All centrifugations were carried out at 4°C. The pelleted membranes were resuspended in binding buffer (50 mM Tris-HCl, 120 mM NaCl, 5 mM KCl, 1 mM MgCl_2_, 2.5 mM CaCl_2_, pH 7.0) and protein concentration determined using the DC Protein Assay kit (Bio-Rad Laboratories, Ltd., Hemel Hempstead, UK). Binding assays were assembled on ice in borosilicate glass test tubes with 50–100 µg membrane protein in binding buffer in a final volume of 250 µl. Binding reactions were incubated at 4°C overnight, and then terminated by rapid vacuum filtration using a Brandel Cell Harvester onto Whatman GF/B glass fibre filters pre-soaked in 0.4% polyethylenimine. Saturation binding experiments were performed with [^125^I]α-BTX concentrations ranging from 0.03–100 nM. Displacement binding experiments were performed with a constant [^125^I]α-BTX concentration of 2.0 nM. Non-specific binding was determined with 10 µM MLA.

### Data Analysis

Ligand binding data were analyzed using GraphPad Prism 4.03 (GraphPad Software, Inc.) Saturation binding data were fitted by nonlinear regression with a hyperbolic function for a one-binding site model. Displacement binding data were fitted by nonlinear regression for a one-site binding competition model, unless otherwise noted. RT-PCR band density data was analysed using Student's t-test for comparison of individual means with control values and by one-way ANOVA followed by Tukey's multiple comparison test for comparison of test groups and effect of MLA inhibition effects.

### Total RNA Isolation, cDNA Preparation and PCR Amplification

Primers for RNA analysis were designed using the Primer3 program [92] and analysed for structural anomalies and dimer formation using NetPrimer software (Premier Biosoft International, Palo Alto, USA). Primer specificity was confirmed by comparison with DNA sequence databases using nucleotide-nucleotide BLAST (Available at http://www.ncbi.nlm.nih.gov). Forward and reverse primers for each gene of interest were designed from separate exon sequences to eliminate possible artefacts due to potential DNA contamination in RNA preps. Primers used were as follows: GAPDH (NM_017008) forward: gaacatcatccctgcatcca, reverse: ccagtgagcttcccgttca; α7-nAChR (NM_00746) forward: ggaagctttacaaggagctg, reverse: gccatctgggaaacgaaca.

Total RNA was isolated from GH4-hα7 cells using the Sigma GenElute™ Mammalian Total RNA kit. RNA was reverse transcribed into cDNA using SuperScript First-Strand Synthesis System (Invitrogen, Paisley, UK) as per the manufacturer's instructions. 100 ng cDNA was amplified by PCR with 50 pmol gene-specific primers, 1.5 mM MgCl_2_, 200 µM dNTPs, and 1.25 U *Taq* DNA polymerase (Promega, Southampton, UK) in a 50 µl final reaction volume. After an initial denaturation of 95°C for 2 min, reactions were amplified for 30 cycles: 95°C for 30 s, 55°C for 30 s, 72°C for 1 min, followed by a final extension of 72°C for 10 min. Reaction products were separated by electrophoresis on 1.5% agarose TAE gels and visualized by UV illumination. Images were captured using a Bio-Rad Gel Doc 2000 and QuantityOne software (Bio-Rad, Hempstead, UK).

### Lysate Preparation, Cellular Fractionation, Protein Evaluation, SDS-PAGE and Western Blot Analysis

After exposure to peptides for indicated times, cells were harvested in 25 mM Tris HCl containing 2 mM EDTA and 1× protease inhibitor cocktail (Roche Diagnostics, Ltd.,West Sussex, UK) and pelleted at 4°C and 10,000×g for 10 minutes. Cell lysates were prepared in lysis buffer containing 25 mM Tris HCl pH 7.4, 150 mM NaCl, 2 mM EDTA, 0.1 mM phenylmethylsulfonyl fluoride, 0.1% Nonidet, and 1× protease inhibitor cocktail. Cells were vortexed vigorously for 5 minutes placing on ice intermittently. After separation of nuclei, ¼ of the cell lysate was kept as total homogenate for further analysis, while the remaining ¾ was used to separate out the membrane fraction. Briefly, cell lysates were pelleted by centrifugation for 50 minutes at 4°C and 100,000×g. The resulting pellets (membranes) were resuspended in lysis buffer and protein levels (α7-nAChR and actin) were determined by western blot analysis. Equal amounts of protein were prepared in Laemmli buffer, separated in 7% SDS-PAGE and blotted onto nitrocellulose membrane. For immuno-detection, the following antibodies were used: anti-α7-nAChR (Santa Cruz Biotechnology, Santa Cruz, CA, USA; dilution 1∶200), anti-actin (Sigma-Aldrich, Poole, Dorset, UK; dilution 1∶5,000), peroxidase-conjugated donkey anti-goat (Sigma-Aldrich, Poole, Dorset, UK; dilution 1∶10,000), and peroxidase-conjugated goat anti-rabbit (Pierce, Rockford, IL, USA, dilution 1∶10,000). Blots were developed with enhanced chemiluminescence (ECL; GE Healthcare, Little Chalfont, Buckinghamshire, UK).

### Immunocytochemistry

GH4-hα7 cells were seeded into 12-well cell culture plates (5×10^4^ cells/well) containing 13 mm glass coverslips pre-coated sequentially with poly-D-lysine (MW 30,000–70,000; 1 mg/ml) and rat tail-derived collagen Type 1A (100 µg/ml). Cells were allowed to recover for 24–48 hours before experimentation. After treating cells with peptides (100 nM) for 24 hr, medium was removed and cells were washed with phosphate-buffered saline (PBS; 10 mM phosphate buffer, pH = 7.4, 150 mM NaCl, 2.7 mM KCl). Cells were fixed with 2% paraformaldehyde in phosphate buffer (100 mM, pH = 7.4), permeablized with 0.1% Triton X-100 in PBS, blocked with 2% bovine serum albumin (BSA) in PBS, and then incubated with goat anti-α7-nAChR (Santa Cruz Biotechnology, Santa Cruz, CA, USA; dilution 1∶100) in 2% BSA/PBS buffer for 1.5 hr. After thorough washing with PBS to remove excess primary antibody, cells were incubated with Alexafluor 488-conjugated donkey anti-goat secondary antibody (1∶800; Molecular Probes) overnight at 4°C. Cells were washed with PBS, and then coverslips were mounted on slides with ProLong Gold® anti-fade mounting medium (Life Technologies Ltd., Paisley, UK). Fluorescent signals were visualized using a Leitz Diaplan microscope and images were captured with a Leica DFC300FX digital camera and Leica DFC Twain imaging software (Leica Microsystems Ltd., Milton Keynes, UK). For assessment of α7-nAChR protein expression, at least 3 coverslips were examined for each control and peptide treatment and a minimum of 12 randomly selected visual fields (40×) were acquired from each coverslip.

## References

[1] Choi DW (1992). Excitotoxic cell death.. J Neurobiol.

[2] Greenfield SA, Vaux DJ (2002). Parkinson's disease, Alzheimer's disease and motor neurone disease: identifying a common mechanism.. Neuroscience.

[3] Salińska E, Danysz W, Łazarewicz JW (2005). The role of excitotoxicity in neurodegeneration.. Folia Neuropathol.

[4] Van Damme P, Dewil M, Robberecht W, Van Den Bosch L (2005). Excitotoxicity and amyotrophic lateral sclerosis.. Neurodegener Dis.

[5] Seguela P, Wadiche J, Dineley-Miller K, Dani JA, Patrick JW (1993). Molecular cloning, functional properties, and distribution of rat brain alpha 7: a nicotinic cation channel highly permeable to calcium.. J Neurosci.

[6] Greenberg ME, Ziff EB, Greene LA (1986). Stimulation of neuronal acetylcholine receptors induces rapid gene transcription.. Science.

[7] Chang KT, Berg DK (2001). Voltage-gated channels block nicotinic regulation of CREB phosphorylation and gene expression in neurons.. Neuron.

[8] Dajas-Bailador F, Wonnacott S (2004). Nicotinic acetylcholine receptors and the regulation of neuronal signalling.. Trends Pharmacol Sci.

[9] Dunckley T, Lukas RJ (2006). Nicotinic modulation of gene expression in SH-SY5Y neuroblastoma cells.. Brain Res.

[10] Wang HY, Lee DH, Davis CB, Shank RP (2000). Amyloid peptide Abeta(1–42) binds selectively and with picomolar affinity to alpha7 nicotinic acetylcholine receptors.. J Neurochem.

[11] Bednar I, Paterson D, Marutle A, Pham TM, Svedberg M (2002). Selective nicotinic receptor consequences in APP(SWE) transgenic mice.. Mol Cell Neurosci.

[12] Bourin M, Ripoli N, Dailly E (2003). Nicotinic receptors and Alzheimer's disease.. Curr Med Res Opin.

[13] Mousavi M, Bednar I, Nordberg A (2004). Selective changes in expression of different nicotinic receptor subtypes in brain and adrenal glands of mice carrying human mutated gene for APP or over-expressing human acetylcholinestrase.. Int J Dev Neurosci.

[14] Chu LW, Ma ES, Lam KK, Chan MF, Lee DH (2005). Increased alpha 7 nicotinic acetylcholine receptor protein levels in Alzheimer's disease patients.. Dement Geriatr Cogn Disord.

[15] Yu WF, Guan ZZ, Bogdanovic N, Nordberg A (2005). High selective expression of alpha7 nicotinic receptors on astrocytes in the brains of patients with sporadic Alzheimer's disease and patients carrying Swedish APP 670/671 mutation: a possible association with neuritic plaques.. Exp Neurol.

[16] Jones IW, Westmacott A, Chan E, Jones RW, Dineley K (2006). Alpha7 nicotinic acetylcholine receptor expression in Alzheimer's disease: receptor densities in brain regions of the APP(SWE) mouse model and in human peripheral blood lymphocytes.. J Mol Neurosci.

[17] Pereira EF, Reinhardt-Maelicke S, Schrattenholz A, Maelicke A, Albuquerque EX (1993). Identification and functional characterization of a new agonist site on nicotinic acetylcholine receptors of cultured hippocampal neurons.. J Pharmacol Exp Ther.

[18] Samochocki M, Höffle A, Fehrenbacher A, Jostock R, Ludwig J (2003). Galantamine is an allosterically potentiating ligand of neuronal nicotinic but not of muscarinic acetylcholine receptors.. J Pharmacol Exp Ther.

[19] Papke RL, Bencherif M, Lippiello P (1996). An evaluation of neuronal nicotinic acetylcholine receptor activation by quaternary nitrogen compounds indicates that choline is selective for the alpha 7 subtype.. Neurosci Lett.

[20] Alkondon M, Pereira EF, Cortes WS, Maelicke A, Albuquerque EX (1997). Choline is a selective agonist of alpha7 nicotinic acetylcholine receptors in the rat brain neurons.. Eur J Neurosci.

[21] Nováková J, Mikasová L, Machová E, Lisá V, Dolezal V (2005). Chronic treatment with amyloid beta(1–42) inhibits non-cholinergic high-affinity choline transport in NG108-15 cells through protein kinase C signaling.. Brain Res.

[22] Payette DJ, Xie J, Guo Q (2007). Reduction in CHT1-mediated choline uptake in primary neurons from presenilin-1 M146V mutant knock-in mice.. Brain Res.

[23] Wang B, Yang L, Wang Z, Zheng H (2007). Amyloid precursor protein mediates presynaptic localization and activity of the high-affinity choline transporter.. Proc Nat Acad Sci U S A.

[24] Greenfield SA (1984). Acetylcholinesterase may have novel functions in the brain.. Trends Neurosci.

[25] Paraoanu LE, Steinert G, Klaczinski J, Becker-Röck M, Bytyqi A (2006). On functions of cholinesterases during embryonic development.. J Mol Neurosci.

[26] Sharma KV, Koenigsberger C, Brimijoin S, Bigbee JW (2001). Direct evidence for an adhesive function in the noncholinergic role of acetylcholinesterase in neurite outgrowth.. J Neurosci Res.

[27] Greenfield SA, Day T, Mann EO, Bermudez I (2004). A novel peptide modulates alpha7 nicotinic receptor responses: implications for a possible trophic-toxic mechanism within the brain.. J Neurochem.

[28] Zbarsky V, Thomas J, Greenfield S (2004). Bioactivity of a peptide derived from acetylcholinesterase: involvement of an ivermectin-sensitive site on the alpha 7 nicotinic receptor.. Neurobiol Dis.

[29] Bon CL, Greenfield SA (2003). Bioactivity of a peptide derived from acetylcholinesterase: electrophysiological characterization in guinea-pig hippocampus.. Eur J Neurosci.

[30] Day T, Greenfield SA (2003). A peptide derived from acetylcholinesterase induces neuronal cell death: characterisation of possible mechanisms.. Exp Brain Res.

[31] Day T, Greenfield SA (2004). Bioactivity of a peptide derived from acetylcholinesterase in hippocampal organotypic cultures.. Exp Brain Res.

[32] Emmett SR, Greenfield SA (2004). A peptide derived from the C-terminal region of acetylcholinesterase modulates extracellular concentrations of AChE in the substantia nigra.. Neurosci Lett.

[33] Onganer PU, Djamgoz MB, Whyte K, Greenfield SA (2006). An acetylcholinesterase-derived peptide inhibits endocytic membrane activity in a human metastatic breast cancer cell line.. Biochim Biophys Acta.

[34] Coleman BA, Taylor P (1996). Regulation of acetylcholinesterase expression during neuronal differentiation.. J Biol Chem.

[35] Day T, Greenfield SA (2002). A non-cholinergic, trophic action of acetylcholinesterase on hippocampal neurons in vitro: molecular mechanisms.. Neuroscience.

[36] Paraoanu LE, Layer PG (2008). Acetylcholinesterase in cell adhesion, neurite growth and network formation.. FEBS J.

[37] Jiang H, Zhang XJ (2008). Acetylcholinesterase and apoptosis. A novel perspective for an old enzyme.. FEBS J.

[38] Park SE, Jeong SH, Yee SB, Kim TH, Soung YH (2008). Interactions of acetylcholinesterase with caveolin-I and subsequently with cytochrome c are required for apoptosome formation.. Carcinogenesis.

[39] Belbeoc'h S, Falasca C, Leroy J, Ayon A, Massoulié J (2004). Elements of the C-terminal t peptide of acetylcholinesterase that determine amphiphilicity, homomeric and heteromeric associations, secretion and degradation.. Eur J Biochem.

[40] Massoulié J, Bon S (2006). The C-terminal T peptide of cholinesterases: structure, interactions, and influence on protein folding and secretion.. J Mol Neurosci.

[41] Jean L, Thomas B, Tahiri-Alaoui A, Shaw M, Vaux DJ (2007). Heterologous amyloid seeding: revisiting the role of acetylcholinesterase in Alzheimer's disease. PLoS ONE 2.. http://www.plosone.org/article/info:doi/10.1371/journal.pone.0000652.

[42] Cheng Y, Prusoff WH (1973). Relationship between the inhibition constant (K1) and the concentration of inhibitor which causes 50 per cent inhibition (I50) of an enzymatic reaction.. Biochem Pharmacol.

[43] Cooper ST, Millar NS (1997). Host cell-specific folding and assembly of the neuronal nicotinic acetylcholine receptor alpha7 subunit.. J Neurochem.

[44] Bond CE, Greenfield SA (2007). Multiple cascade effects of oxidative stress on astroglia.. Glia.

[45] Albert PR, Tashjian AH (1984). Relationship of thyrotropin-releasing hormone-induced spike and plateau phases in cytosolic free Ca2+ concentrations to hormone secretion.. J Biol Chem.

[46] Williams ME, Burton B, Urrutia A, Shcherbatko A, Chavez-Noriega LE (2005). Ric-3 promotes functional expression of the nicotinic acetylcholine receptor alpha7 subunit in mammalian cells.. J Biol Chem.

[47] Millar NS (2008). RIC-3: a nicotinic acetylcholine receptor chaperone.. Br J Pharm.

[48] Taylor P, Radić Z, Kreienkamp HJ, Maeda R, Luo Z (1994). Expression and ligand specificity of acetylcholinesterase and the nicotinic receptor: a tale of two cholinergic sites.. Biochem Soc Trans.

[49] Broide RS, Robertson RT, Leslie FM (1996). Regulation of alpha7 nicotinic acetylcholine receptors in the developing rat somatosensory cortex by thalamocortical afferents.. J Neurosci.

[50] Torrãoa AS, Carmonaa FMM, Lindstromb J, Britto LRG (2000). Expression of cholinergic system molecules during development of the chick nervous system.. Dev Brain Res.

[51] Liu Z, Zhang J, Berg DK (2007). Role of endogenous nicotinic signaling in guiding neuronal development.. Biochem Pharmacol.

[52] Zhang XJ, Yang L, Zhao Q, Caen JP, He HY (2002). Induction of acetylcholinesterase expression during apoptosis in various cell types.. Cell Death Differ.

[53] Hruska M, Nishi R (2007). Cell-autonomous inhibition of alpha 7-containing nicotinic acetylcholine receptors prevents death of parasympathetic neurons during development.. J Neurosci.

[54] Dineley KT, Westerman M, Bui D, Bell K, Ashe KH (2001). Beta-amyloid activates the mitogen-activated protein kinase cascade via hippocampal alpha7 nicotinic acetylcholine receptors: In vitro and in vivo mechanisms related to Alzheimer's disease.. J Neurosci.

[55] Fodero LR, Mok SS, Losic D, Martin LL, Aguilar MI (2004). Alpha7-nicotinic acetylcholine receptors mediate an Abeta(1–42)-induced increase in the level of acetylcholinesterase in primary cortical neurones.. J Neurochem.

[56] Fossier P, Baux G, Tauc L (1983). Possible role of acetylcholinesterase in regulation of postsynaptic receptor efficacy at a central inhibitory synapse of *Aplysia*.. Nature.

[57] Sternfeld M, Shohami S, Klein O, Flores-Flores C, Evron T (2000). Excess “read-through” acetylcholinesterase attenuates but the “synaptic” variant intensifies neurodeterioration correlates.. Proc Natl Acad Sci U S A.

[58] Rees TM, Berson A, Sklan EH, Younkin L, Younkin S (2005). Memory deficits correlating with acetylcholinesterase splice shift and amyloid burden in doubly transgenic mice.. Curr Alzheimer Res.

[59] Jin QH, He HY, Shi YF, Lu H, Zhang XJ (2004). Overexpression of acetylcholinesterase inhibited cell proliferation and promoted apoptosis in NRK cells.. Acta Pharmacol Sin.

[60] Alvarez A, Alarcón R, Opazo C, Campos EO, Muñoz FJ (1998). Stable complexes involving acetylcholinesterase and amyloid-beta peptide change the biochemical properties of the enzyme and increase the neurotoxicity of Alzheimer's fibrils.. J Neurosci.

[61] Berson A, Knobloch M, Hanan M, Diamant S, Sharoni M (2008). Changes in readthrough acetylcholinesterase expression modulate amyloid-beta pathology.. Brain.

[62] Inestrosa NC, Dinamarca MC, Alvarez A (2008). Amyloid-cholinesterase interactions. Implications for Alzheimer's disease.. FEBS J.

[63] Schwarz M, Loewenstein-Lichtenstein Y, Glick D, Liao J, Norgaard-Pedersen B (1995). Successive organophosphate inhibition and oxime reactivation reveals distinct responses of recombinant human cholinesterase variants.. Brain Res Mol Brain Res.

[64] Massoulié J (2002). The origin of the molecular diversity and functional anchoring of cholinesterases.. Neurosignals.

[65] Dori A, Soreq H (2006). ARP, the cleavable C-terminal peptide of “readthrough” acetylcholinesterase, promotes neuronal development and plasticity.. J Mol Neurosci.

[66] Sternfeld M, Ming G, Song H, Sela K, Timberg R (1998). Acetylcholinesterase enhances neurite growth and synapse development through alternative contributions of its hydrolytic capacity, core protein, and variable C termini.. J Neurosci.

[67] Saxena A, Hur RS, Luo C, Doctor BP (2003). Natural monomeric form of fetal bovine serum acetylcholinesterase lacks the C-terminal tetramerization domain.. Biochemistry.

[68] Evron T, Greenberg D, Mor TS, Soreq H (2007). Adaptive changes in acetylcholinesterase gene expression as mediators of recovery from chemical and biological insults.. Toxicology.

[69] Santos SC, Vala I, Miguel C, Barata JT, Garção P (2007). Expression and subcellular localization of a novel nuclear acetylcholinesterase protein.. J Biol Chem.

[70] Anderson AA, Ushakov DS, Ferenczi MA, Mori R, Martin P (2007). Morphoregulation by acetylcholinesterase in fibroblasts and astrocytes.. J Cell Physiol.

[71] Cottingham MG, Voskuil JL, Vaux DJ (2003). The intact human acetylcholinesterase C-terminal oligomerization domain is alpha-helical in situ and in isolation, but a shorter fragment forms beta-sheet-rich amyloid fibrils and protofibrillar oligomers.. Biochemistry.

[72] Papke RL, Meyer E, Nutter T, Uteshev VV (2000). Alpha7 receptor-selective agonists and modes of alpha7 receptor activation.. Eur J Pharmacol.

[73] Zwart R, van Kleef RG, Gotti C, Smulders CJ, Vijverberg HP (2000). Competitive potentiation of acetylcholine effects on neuronal nicotinic receptors by acetylcholinesterase-inhibiting drugs.. J Neurochem.

[74] Pereira EF, Hilmas C, Santos MD, Alkondon M, Maelicke A (2002). Unconventional ligands and modulators of nicotinic receptors.. J Neurobiol.

[75] Liu Q, Kawai H, Berg DK (2001). beta -Amyloid peptide blocks the response of alpha 7-containing nicotinic receptors on hippocampal neurons.. Proc Natl Acad Sci U S A.

[76] Pettit DL, Shao Z, Yakel JL (2001). beta-Amyloid(1–42) peptide directly modulates nicotinic receptors in the rat hippocampal slice.. J Neurosci.

[77] Lukas RJ, Bennett EL (1980). Chemical modification and reactivity of sulfhydryls and disulfides of rat brain nicotinic-like acetylcholine receptors.. J Biol Chem.

[78] Hamouda AK, Chiara DC, Sauls D, Cohen JB, Blanton MP (2006). Cholesterol interacts with transmembrane alpha-helices M1, M3, and M4 of the Torpedo nicotinic acetylcholine receptor: photolabeling studies using [3H]Azicholesterol.. Biochemistry.

[79] Blanton MP, Xie Y, Dangott LJ, Cohen JB (1999). The steroid promegestone is a noncompetitive antagonist of the Torpedo nicotinic acetylcholine receptor that interacts with the lipid-protein interface.. Mol Pharmacol.

[80] Arias HR, Bhumireddy P, Bouzat C (2006). Molecular mechanisms and binding site locations for noncompetitive antagonists of nicotinic acetylcholine receptors.. Int J Biochem Cell Biol.

[81] Uteshev VV, Meyer EM, Papke RL (2003). Regulation of neuronal function by choline and 4OH-GTS-21 through alpha 7 nicotinic receptors.. J Neurophysiol.

[82] Allen DD, Galdzicki Z, Brining SK, Fukuyama R, Rapoport SI, Smith QR (1997). Beta-amyloid induced increase in choline flux across PC12 cell membranes.. Neurosci Lett.

[83] Jonnala RR, Buccafusco JJ (2001). Relationship between the increased cell surface alpha7 nicotinic receptor expression and neuroprotection induced by several nicotinic receptor agonists.. J Neurosci Res.

[84] Kawai H, Berg DK (2001). Nicotinic acetylcholine receptors containing alpha 7 subunits on rat cortical neurons do not undergo long-lasting inactivation even when up-regulated by chronic nicotine exposure.. J Neurochem.

[85] Sallette J, Pons S, Devillers-Thiery A, Soudant M, Prado de Carvalho L (2005). Nicotine upregulates its own receptors through enhanced intracellular maturation.. Neuron.

[86] Kalamida D, Poulas K, Avramopoulou V, Fostieri E, Lagoumintzis G (2007). Muscle and neuronal nicotinic acetylcholine receptors. Structure, function and pathogenicity.. FEBS J.

[87] Ochoa EL, Chattopadhyay A, McNamee MG (1989). Desensitization of the nicotinic acetylcholine receptor: molecular mechanisms and effect of modulators.. Cell Mol Neurobiol.

[88] Hall RA, Soderling TR (1997). Differential surface expression and phosphorylation of the N-methyl-D-aspartate receptor subunits NR1 and NR2 in cultured hippocampal neurons.. J Biol Chem.

[89] Kay BK, Williamson MP, Sudol M (2000). The importance of being praline: the interaction of praline-rich motifs in signaling proteins with their cognate domains.. FASEB J.

[90] Venot C, Maratrat M, Dureuill C, Conseiller E, Bracco L (1998). The requirement for the p53 proline-rich functional domain for mediation of apoptosis is correlated with specific *PIG3* gene transactivation and with transcriptional repression.. EMBO J.

[91] Svedberg MM, Svensson AL, Johnson M, Lee M, Cohen O (2002). Upregulation of neuronal nicotinic receptor subunits alpha4, beta2, and alpha7 in transgenic mice overexpressing human acetylcholinesterase.. J Mol Neurosci.

[92] Rozen S, Slaletsky H, Krawetz S, Misener S (2000). Primer3 on the WWW for general users and for biological programmers.. Bioinformatics methods and protocols: Methods in molecular biology.

